# Exposing Deep Representations to a Recurrent Expansion with Multiple Repeats for Fuel Cells Time Series Prognosis

**DOI:** 10.3390/e24071009

**Published:** 2022-07-21

**Authors:** Tarek Berghout, Mohamed Benbouzid, Toufik Bentrcia, Yassine Amirat, Leïla-Hayet Mouss

**Affiliations:** 1 Laboratory of Automation and Manufacturing Engineering, University of Batna 2, Batna 05000, Algeria; t.berghout@univ-batna2.dz (T.B.); t.bentrcia@univ-batna2.dz (T.B.); h.mouss@univ-batna2.dz (L.-H.M.); 2 Institut de Recherche Dupuy de Lôme (UMR CNRS 6027), University of Brest, 29238 Brest, France; 3 Logistics Engineering College, Shanghai Maritime University, Shanghai 201306, China; 4 ISEN Yncréa Ouest, L@bISEN, 29200 Brest, France; yassine.amirat@isen-ouest.yncrea.fr

**Keywords:** deep learning, fuel cell, long short-term memory, recurrent expansion, remaining useful life, prognosis and health management

## Abstract

The green conversion of proton exchange membrane fuel cells (PEMFCs) has received particular attention in both stationary and transportation applications. However, the poor durability of PEMFC represents a major problem that hampers its commercial application since dynamic operating conditions, including physical deterioration, have a serious impact on the cell performance. Under these circumstances, prognosis and health management (PHM) plays an important role in prolonging durability and preventing damage propagation via the accurate planning of a condition-based maintenance (CBM) schedule. In this specific topic, health deterioration modeling with deep learning (DL) is the widely studied representation learning tool due to its adaptation ability to rapid changes in data complexity and drift. In this context, the present paper proposes an investigation of further deeper representations by exposing DL models themselves to recurrent expansion with multiple repeats. Such a recurrent expansion of DL (REDL) allows new, more meaningful representations to be explored by repeatedly using generated feature maps and responses to create new robust models. The proposed REDL, which is designed to be an adaptive learning algorithm, is tested on a PEMFC deterioration dataset and compared to its deep learning baseline version under time series analysis. Using multiple numeric and visual metrics, the results support the REDL learning scheme by showing promising performances.

## 1. Introduction

**Figure 1:**
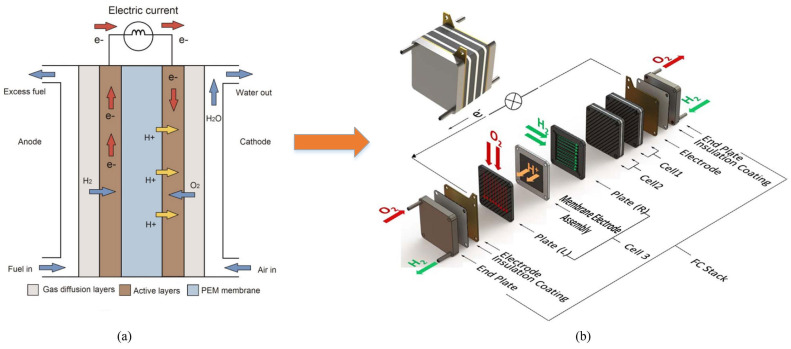
Schematic diagram of PEMFC stack and operating principle: (**a**) operating principle of a PEMFC. Reprinted with permission from Ref. [2], Wiley Online Library: 2017; (**b**) PEMFCs stack structure. Reprinted with permission from Ref. [3], Elsevier: 2017.

Recent prospective studies have revealed that over the next 30 years, the expected demographic rise in the world’s population will increase energy demand by 50% [1]. As a result, and among the different types of fuel cells, PEMFCs are considered to be the most feasible green energy converter, suitable for both stationary and transportation applications [2]. PEMFC appearing on the schematic diagram of Figure 1a is intended to convert chemical energy into electricity from hydrogen fuel. It consists of an anode, a cathode, and an electrolyte. The electrolyte is a non-conductive material that transmits charges from the cathode to the anode via an external circuit [3]. Generally, a single PEMFC can be considered a lower voltage and higher current electricity generator that ranges from 0.4 V to 0.9 V and from 0.5 A/cm^2^ to 1 A/cm^2^, respectively. Therefore, as shown in Figure 1b, several fuel cells must be stacked to satisfy a specific type of application [3]. It is very common for PEMFC to be highly sensitive to dynamic and non-stationary external and internal operating conditions, which could reduce its lifespan [4]. In this context, components material (Figure 1), gas contamination, and the operating conditions imposed by the load profile are responsible for the system durability. Accordingly, a well-structured prognosis policy is needed to extend the lifetime by incorporating the required CBM tasks [5].

Indeed, the remaining useful life (RUL) prediction, which is the estimation of the time between the current and the end of life of a system, is a key element for assessing damage propagation and aging [6,7]. Two particular types of prognosis can be triggered in this case: the short-term prognosis and the long-term prognosis [4,8]. Short-term prognosis is designed to capture local variations with high accuracy, while long-term prognosis aims to give the deterioration trend [8]. Logically, the prediction of local variations will be more accurate in comparison to the large variation of the trend of the life path. For PEMFCs, prognosis can be modeled through three main strategies, namely model-based, filter-based, and data-based approaches [9]. Among data-based approaches, DL tools have been widely investigated. According to [5], the reason for choosing DL tools is motivated by the need to provide a meaningful representation of data patterns originally presented in a very complex feature space (see Figure 2 from [5]). In the context of PEMFCs prognosis, the entire deterioration path can be obtained under real experimental conditions. As a result, data will be “*complete*” in this case. Additionally, it is common for PEMFCs to be subject to a higher level of dynamic disturbances caused by changing operating conditions leading to more “*complex*” and “*drifted*” data. If we project these three characteristics (i.e., data is complete, complex, and drifted) onto the flowchart proposed in the systematic guide of [5] (see Figure 2 of [5]), there would be no more optimal solution than DL for such a case, and conventional machine learning will no longer be recommended. Thus, reconstructing an RUL predictive model requires meeting three main criteria: data *availability*, *complexity*, and *drift*. In this context, to remedy learning issues regarding adaptive learning, dynamic programming is highly recommended to strengthen the learning model. Additionally, to overcome data complexity issues resulting from changing operating conditions, DL is also the main recommended path.

Among recently published papers dealing with DL in the field of performance evaluation of PEMFCs including prognosis (i.e., RUL prediction), learning techniques, which have been derived from leading supervised learning tools such as convolutional neural networks (CNN) [10,11,12,13], long short-term memory (LSTM) [14,15,16], deep belief neural networks (DBNs), and autoencoders (AEs) [17,18] have been extensively investigated. CNNs are generally recommended when trying to separate between different data patterns and make sure they offer a quite enhanced representation. However, CNNs’ learning rules as originally proposed lack the element of adaptive learning. Contrariwise, LSTMs and their variants such as gated units are very popular adaptive learning and time series networks [19]. Unlike CNNs, their robustness resembled each other in considering the mathematical correlation between samples behaving similarly. At the same time, adaptive learning baselines intend to ignore (i.e., forgetting) unwanted learning samples depending on specific parameters known as gates. In the meanwhile, DBNs are totally dependent on the robustness of feature extractions. In this case, unsupervised generative models, such as autoencoders and even adversarial networks, could be involved in fine-tuning DBNs for supervised learning.

Generally speaking, the above-discussed learning models are designed to achieve real-time performance evaluation. More specifically for RUL prediction models, they have been designed either for a single step-ahead or multistep-ahead prediction. These training scenarios have been realized under previously discussed prognosis types including either a long-term or a short-term prognosis. Depending on the type of training rules, these models seemed to be successful regressors in terms of dynamic programming, approximation, or generalization. In this context, the main open challenge, in this case, is to provide a better representation than these DL models to the feature spaces. This is conducted to ensure exploring more meaningful patterns in data, which logically yields better performances.

In this context, the contributions of this paper are threefold:

- In an attempt to address adaptive learning, the LSTM learning rules that seem to be a perfect choice in this case are selected to train the DL model;
- To explore new representations learning features, the LSTM network has been exposed to a recurrent expansion process with multiple repeats. This process will not make the LSTM learn from input representation only, but it will also make it able to learn from feature maps and estimated targets. This makes a total of three sources of information, which are repeatedly merged into several LSTMs, leading to a deeper representation than ordinary LSTMs;
- To evaluate the REDL model, the well-known PHM 2014 data challenge dataset has been employed under a long-term prognosis based on a single step-ahead prediction.

This paper is organized as follows: Section 2 is dedicated to the problem description. In Section 3, we introduce the REDL model and its learning rules. Section 4 is devoted to experiments and results discussion. Section 5 concludes this study with some remarks.

## 2. Problem Description

**Figure 2:**
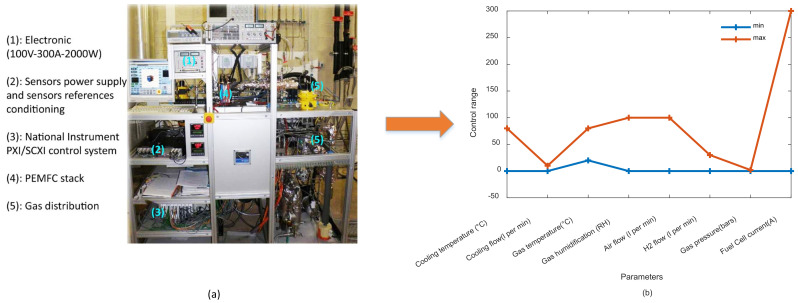
FCLAB test bench and operating conditions: (**a**) FCLAB test bench. Reprinted with permission from Ref. [19], MDPI: 2022; (**b**) operating conditions limits.

Data investigated in this study is provided by the fuel cell laboratory (FCLAB FR CNRS 3539, France, http://eng.fclab.fr/ accessed on 7 June 2022), where it was first introduced at the IEEE PHM data challenge in 2014 [20]. The FCLAB test bench allows performing experimental studies for both ordinary and accelerated degradations, whereas it also allows access to monitoring parameters (e.g., power loads, temperatures, hydrogen and air stoichiometry rates, etc.) and modifying operating conditions. The test bench in Figure 2a is mainly used to study FCs with a maximum power of 1 kW, while operating conditions are depicted in Figure 2b. Gas humidification and the transport of air and hydrogen are ensured by specific independent boilers placed upstream of the FC stack, where only the air boiler is subject to heat control to achieve the desired relative humidity. The hydrogen boiler has an ambient temperature controller, whilst the stack temperature and power supply are controlled by cooling water and a TDI Dynaload active load, respectively. The studied PEMFC stack contains five cells with an active surface of 100 cm^2^. The PEMFC is constructed with commercial membranes, diffusion layers, and machined flux distribution plates. The PEMFC can reach a nominal current density of the cells equal to 0.70 A/cm^2^ while the maximum current density is 1 A/cm^2^.

During this experiment, two long-term durability tests are carried out. The first experiment (FC1) is mainly dedicated to the stationary regime where the operating conditions are considered constant and stable during the whole experiment. The second one (FC2) is mainly focused on the non-stationary regime, where the operating conditions could be subject to a wide range of disturbances. Two types of data were collected, in particular, polarization curves and aging data. Polarization curves are intended to be used for state of health assessment while aging data are used for RUL prediction. In this case, we are interested in aging data as our main goal is to predict RUL.

For FC1 experiment, a complete characterization was achieved every week at times = 0; 48; 185; 348; 515; 658; 823; and 991 h where stationary conditions of a current of 70 A were imposed. First, electrochemical impedance spectroscopy (EIS) was performed only at 0.70 A/cm^2^ for evaluating the FC state before measuring the polarization. After that, the polarization curve was carried out under a current ramp from 0 A/cm^2^ to 1 A/cm^2^ for 1000 s. The air and hydrogen flows were reduced to a current of 20 A where they remained constant at this stage. Finally, the EIS as performed again for constant currents of 0.70 A/cm^2^, 0.45 A/cm^2^, and 0.20 A/cm^2^, respectively, with a stabilization period of 15 min. For the FC2 experiment, the current was subject to dynamic solicitations with a ripple of 70 A, oscillations of 7 A, and a frequency of 5 kHz. Weekly characterization for first polarization curve and EIS, respectively, at times t = 0; 35; 182; 343; 515; 666; 830; and 1016 h were performed.

The collected aging dataset features are defined as follows: Time Aging (time (h)), Single cells and stack voltage (V) (U1–U5 and Utot), Current (A) and current density (A/cm^2^) (I; J), Inlet and Outlet temperatures of H2 (°C) (TinH2; ToutH2), Inlet and Outlet temperatures of Air (°C) (TinAIR; ToutAIR), Inlet and Outlet temperature of cooling Water (°C) (TinWAT; ToutWAT), Inlet and Outlet Pressure of H2 (mbara) (PinH2; PoutH2), Inlet and Outlet Pressure of Air (mbara) (PinAIR; PoutAIR), Inlet and Outlet flow rate of H2 (l/mn) (DinH2; DoutH2), Inlet and Outlet flow rate of Air (l/mn) (DinAIR; DoutAIR), Flow rate of cooling water (l/mn) (DWAT), Inlet Hygrometry (Air) (%) (HrAIRFC). In this case, when considering aging data for RUL prediction, the PHM 2014 main goal was to use the dataset generated from FC1 to reconstruct the data-driven model. After that, the model will be tested on a portion of FC2 experiment, whereas a final step of validation will be performed for long-term predictions using predicted samples themselves.

**Figure 3:**
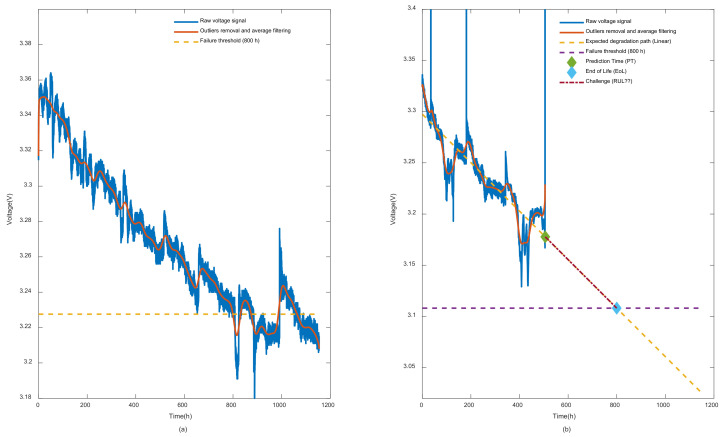
Illustration of the prediction problems main features: (**a**) raw and prepared version of FC1 voltage data for training; (**b**) raw and prepared version of FC2 data for testing and main challenge.

In our work, among a set of previously stated features, the fuel cell voltage, which is a commonly used static health indicator at the cell/battery level, was selected to conduct our time series prognosis experiments. In this context, and since data collection with sensors is subject to outliers, a process of filtration and outliers removal is mandatory. In this case, Figure 3 is introduced to give a better explanation of the RUL prediction problem. In Figure 3a, which represents FC1 case study, the entire PEMFC deterioration patterns are given while only 500 h are revealed for FC2. By comparing FC2 and FC1 voltage behavior, it seems that FC2 is subject to outliers and dynamic disturbance more than FC1. Therefore, a second filtered version of the signal is necessary. In this case, an average window filtering and outlier removers are involved to produce the clean version of the signals as appeared in the figure. In our study, we have set the failure threshold at 800 h as a default value, which reflects about 10% of the voltage drop. It should be mentioned that identifying the failure threshold for PEMFC is still an open question. However, in this situation, the 10% voltage drop is inspired by the United States Department of Energy policy for vehicle applications [1]. In FC2, deterioration patterns from 500 to the failure threshold represent the main challenge of long-term prognosis in this case.

It should be mentioned that the linear degradation path presented in Figure 3b is not obtained by time series predictions based on nonlinear degradation signals. In fact, the time record is used as the inputs of the linear regression model while the filtered signal is the output. This should logically not be the standard way for nonlinear prediction as in our case because time has nothing to do with the cell deterioration. It is, in fact, the operating conditions that have a real effect on the cell as time evolves. However, in our case, the main goal was to create a reference to our predictions so that at least we could assess how far the predictions approximately were away from the degradation trend, especially in the challenge part where the actual data (i.e., truth labels) were not revealed.

**Figure 4:**
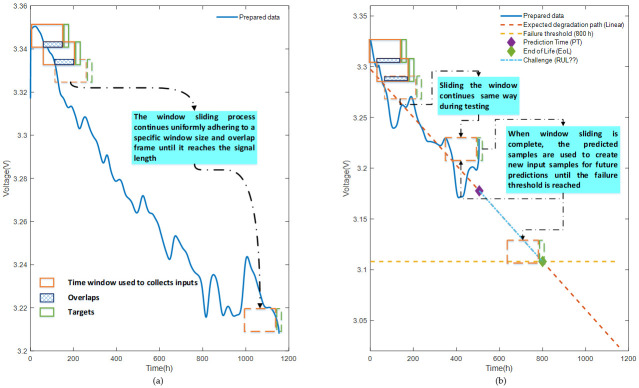
Sliding a time window for samples collection: (**a**) collecting training samples; (**b**) collecting samples of both testing and challenge part.

Accordingly, FC1 data were used to train the prediction model for single-step ahead prediction. While doing so, the FC2 data was used to test the model accuracy. Finally, the challenge part was the prediction using predicted samples from FC2 as an input to the model. To be able to make this work on a time series analysis, a sliding window with a size of 50 samples and an overlap of 30 samples was adopted in this work. Figure 4 clearly indicates the process of data inputs and targets collection for the training (Figure 4a), testing, and challenge parts (Figure 4b).

A very interesting point to be taken into account is that in such particular time series analysis, min–max normalization will no longer be recommended. Especially under single step ahead prediction, samples actual meaning could be easily distorted. Therefore, normalization based on the mean and variance of the entire time frame at each sliding is important and has more significance. We introduce the formula one as the main scaling method recommended in this case. x and $x^{\prime }$ are original and normalized inputs of a single time frame, respectively, while μ and δ refer to mean and standard deviation of the entire training, testing, or challenge dataset.

(1) $$x^{\prime }=\frac{x-\mu }{\delta }$$

## 3. Recurrent Expansion of Deep Learning

**Figure 5:**
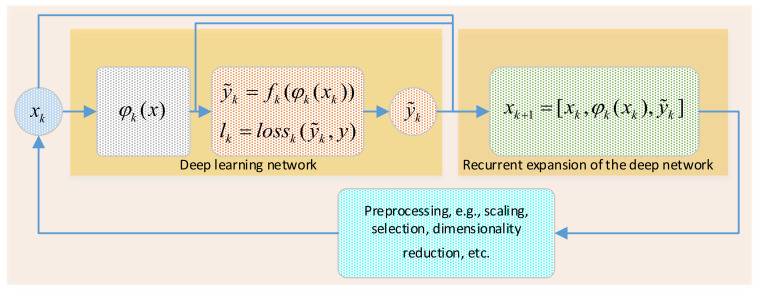
Schematic diagram illustrating REDL algorithm architecture.

This section is introduced to describe the proposed REDL algorithm and its main learning rules. Figure 5 indicates that training a deep network with REDL rules should be performed in accordance with the following steps.

**Step 1**: A deep network must be used to train an approximation function $f_{k}$ in (2) with deep representations $\varphi_{k}\left(x_{k}\right)$ learned from inputs x according to a specific loss function $l_{k}$ in (3). In this case, $\varphi_{k}\left(x_{k}\right)$ could be any sort of feature mapping resulting from training the deep network such as LSTM layers, convolutional mappings, encoded layers of any type of autoencoder, hidden layers from a deep belief network, etc.

(2) $$f_{k}=\varphi_{k}\left(x_{k}\right)$$

(3) $$l_{k}=loss_{k}(y,{\tilde{y}}_{k})$$

**Step 2**: The entire deep network including x*,*$\varphi_{k}\left(x_{k}\right)$, and estimated targets $\tilde{y}$ will be fused in a sort of concatenation as in (4) to train the same type of model repeatedly. In this case, $x_{k+1}$ represents the new inputs to the next training network. k denotes the number of rounds used to repeat the recurrent expansion process.

(4) $$x_{k+1}=[x_{k},\varphi_{k}\left(x_{k}\right),{\tilde{y}}_{k}]$$

**Step 3**: It should be mentioned that the combination in Equation (4) will be extremely huge due to the large layers of the deep network. Moreover, feature maps and estimated targets will no longer follow the same normalization procedures of inputs. Hence, the dimensionality reduction and renormalization of the entire collection is a very important task in this case.

**Step 4**: Stopping criteria, in this case, will be evaluated by different quality approximation metrics depending on the type of the application (i.e., classification or regression). Moreover, the loss function behaviors such as convergence speed and stabilization can be also used to monitor the process of multiple repeat training.

Since the RUL prediction problems are regression ones, well-known quality measuring metrics such as root mean squared error (*RMSE*), mean absolute error (*MAE*), and mean absolute percentage error (*MAPE*) expressed by Formulas (5)–(7), are used in this work. Additionally, for the loss function $l_{k}$, we use also RMSE as the main approximation error. As a result, the area under loss curve (*AULC*) is adopted to indicate the performance of the loss function (8). n in this case refers to the number of training, testing, or challenge samples. m designates the maximum number of epochs.

(5) $$RMSE_{k}=\frac{1}{n}\sum_{i=1}^{n}\sqrt{{\left({\tilde{y}}_{k\left(i\right)}-y_{k\left(i\right)}\right)}^{2}}$$

(6) $${\mathrm{MAE}}_{k}=\frac{1}{n}\sum_{i=1}^{n}\left|{\tilde{y}}_{k\left(i\right)}-y_{k\left(i\right)}\right|$$

(7) $$MAPE_{k}=\frac{1}{n}\sum_{i=1}^{n}\left|\frac{{\tilde{y}}_{k\left(i\right)}-y_{k\left(i\right)}}{y_{k\left(i\right)}}\right|$$

(8) $$AULC_{k}=\int_{0}^{m}l_{k}dx$$

Another interesting scoring metric suggested initially by the PHM 2014 benchmark developers is also proposed. The score functions $S_{k}$ in Equation (9) are used to measure the precision of the prediction model by considering early and late predictions’ effects on maintenance planning. Early predictions have an impact on consuming CBM resources, while later predictions could result in many damages including financial and human losses. $E_{k}$ is a percentage error, a=0.5 is a constant, and b is a penalization parameter given differently for each type of prediction (i.e., b = 5 for late predictions and b = 20 for early ones).

(9) $$S_{k}=e^{ln\left(a\right)\left(E_{k}/b\right)}$$

(10) $$E_{k}=100\%\;\left(\frac{y_{k\left(i\right)}-{\tilde{y}}_{k\left(i\right)}}{y_{k\left(i\right)}}\right)$$

Based on our aforementioned remark regarding the alteration of PEMFC owing to dynamism in operating conditions, the single-layer LSTM was adopted as the main deep architecture to train the REDL model. Accordingly, LSTM layers will represent $\varphi_{k}\left(x_{k}\right)$ and its estimated targetsin each round will be expressed by ${\tilde{y}}_{k}$.

For a better understanding of the followed training procedures, the pseudo-code of Algorithm 1 gives more explanations about REDL training rules.
**Algorithm 1.** REDL algorithm**Inputs:** x,y,k,m,n**Outputs:** 
${\tilde{y}}_{k}$
**For**j=1:k% Train the deep network and evaluate the loss function$f_{k}=\varphi_{k}\left(x_{k}\right)$$l_{k}=loss_{k}(y,{\tilde{y}}_{k})$% Rebuild the new inputs$x_{k+1}=[x_{k},\varphi_{k}\left(x_{k}\right),{\tilde{y}}_{k}]$% Evaluate training metrics$RMSE_{k}=\frac{1}{n}\overset{n}{\underset{i=1}{\sum }}\sqrt{{\left({\tilde{y}}_{k\left(i\right)}-y_{k\left(i\right)}\right)}^{2}}$$MAE_{k}=\frac{1}{n}\overset{n}{\underset{i=1}{\sum }}\left|{\tilde{y}}_{k\left(i\right)}-y_{k\left(i\right)}\right|$$MAPE_{k}=\frac{1}{n}\overset{n}{\underset{i=1}{\sum }}\left|\frac{{\tilde{y}}_{k\left(i\right)}-y_{k\left(i\right)}}{y_{k\left(i\right)}}\right|$$AULC_{k}=\int_{0}^{m}l_{k}dx$$S_{k}=e^{ln\left(a\right)\left(E_{k}/b\right)}$**End (For)**

## 4. Results and Discussion

Prognosis model reconstruction experiments were carried out in MATLAB r2018b environment while computational resources involved a Personal Computer (PC) with an i7 microprocessor, 16 GB of RAM memory, and a 12 MB of cache RAM. A single layer LSTM network with 16 neurons, maximum epochs=300, m=200, minibatch size=150, ADAM optimizer, initial learning rate=0.1, gradient threshold=1, and $L_{2}\;\mathrm{regularization}\;\mathrm{parameter}=0.01$ was trained according to REDL rules. Since our goal, in this case, was to study the effect of REDL rules in improving DL representation for RUL prediction of PEMFC, these parameters were manually tuned according to expert decisions and then fixed for the entire procedure. Additionally, a random seed of learning weights generation was fixed to a single source to make sure that expressed variations in outcomes only resulted from REDL rules and not from other sources. In this context, the LSTM as trained for k=20 rounds with REDL rules.

**Figure 6:**
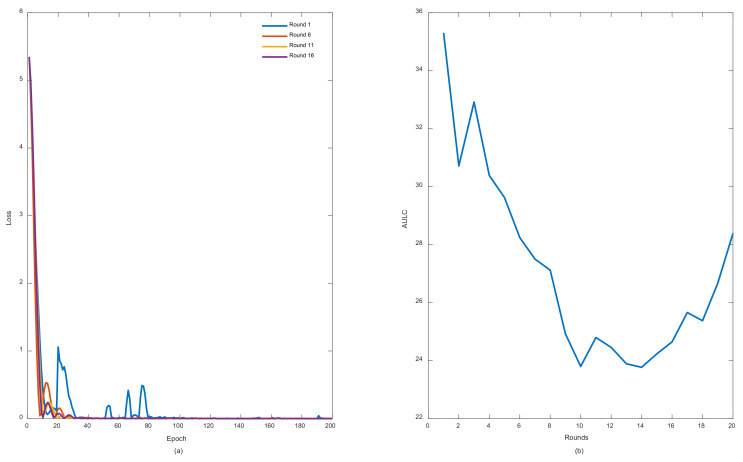
REDL convergence features: (**a**) loss curves during different training rounds; (**b**) AULC values during entire training process.

To evaluate our proposed model, all previously discussed metrics for the entire three subsets including, training, testing, and challenge subsets were recorded in each round. Exceptions were made for the loss function curves and AULC metrics, which were basically related to training only. Figure 6a elucidates some loss curves during those rounds, while Figure 6b illustrates the AULC parameter. Figure 6a shows that in each round from rounds 1 to 16, the loss curves demonstrate less fluctuations, better convergence, and stabilization performances. Figure 6b also reveals the same behavior by highlighting an obvious AULC reduction after each round until a certain threshold. In this case, it can be stated that REDL helps in discovering new representations from each previously trained model. These representations are considered very important until they start to vanish. At this point, unrolling the model several times becomes meaningless to the learning model.

**Figure 7:**
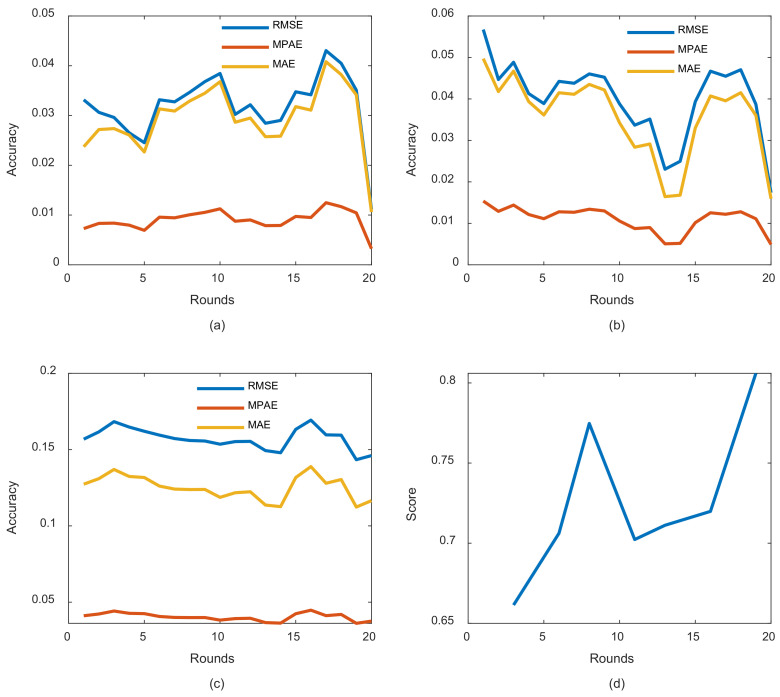
REDL model performances during both training and evaluation: (**a**) REDL training performances in each round; (**b**) REDL testing performances in each round; (**c**) REDL performance at challenge part; (**d**) score behavior during testing phase.

The curves of Figure 7 are also devoted to addressing other metrics in the entire model training and evaluation phases. We can observe significant enhancements in each training round. Exceptions also are made for some rounds, such as in rounds 17 and 18 (from Figure 7a–c). The same previous explanation associated with Figure 6 can be applied here to justify such a phenomenon. Concerning Figure 7d related to the challenge part, the model expresses great performances, which are most important in this case because the main goal is to achieve a long-term prognosis that is as accurate as possible.

**Figure 8:**
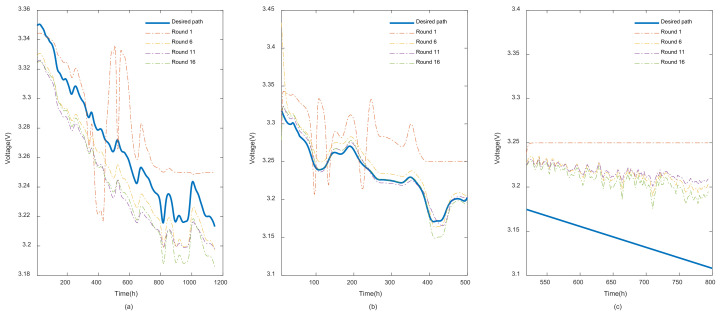
Curve fit results in different rounds: (**a**) REDL curve fit in training phase; (**b**) REDL testing phase; (**c**) REDL long-term predictions.

When it comes to prediction illustrations, REDL curves in different rounds are showcased in Figure 8. It also explains the same previous conclusions where the model in each round clearly reflects a better curve fit. In this case, the proposed score function can be applied as the best way to evaluate the precision of the predictions.

**Figure 9:**
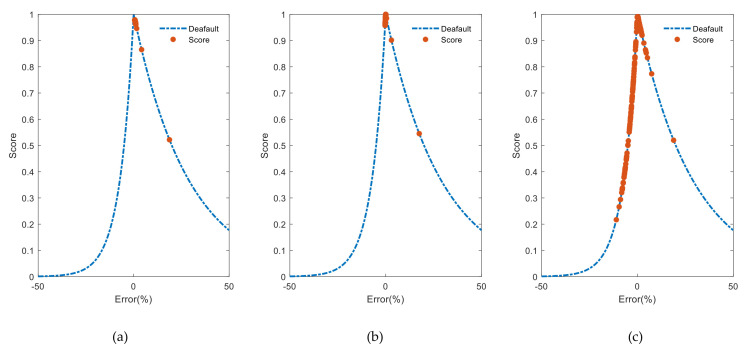
Scoring results in last round: (**a**) scoring results in training phase; (**b**) scoring results in testing phase; (**c**) scoring results in long-term predictions phase.

Figure 9 depicts score values distribution according to the percentage error at the last round. During training and testing phase (Figure 9a,b), the model discloses a high precision when it approaches maximum value one. Moreover, we observe in this case a sort of calibration between early (i.e., E>0) and late predictions (i.e., E<0). Additionally, according to Table 1, the long-term predictions (Figure 9c) are late predictions with a lower precision than the testing phase with about 30%.

Table 1 addresses all obtained results at different phases of model training and the evaluation as well. It is provided that the higher performances of the model are obtained at the last rounds of each phase. Despite the difficulty of obtaining a convincing explanation, it can be asserted that the new training sources including feature maps, and estimated targets from different models have an important role in representation learning compared to a single source of inputs.

## 5. Conclusions

In this paper, we proposed the REDL algorithm as a novel approach for training deep networks when applied to time series prognosis of PEMFCs. The REDL approach is based on improving representation learning features by involving a recurrent expansion rule. The REDL encompasses fusing the entire deep networks including inputs, feature mappings, and estimated targets into other deep learning models with the same architectures for multiple repeats. Different types of visual and numerical metrics have been used during the learning and evaluation processes. A 20-roundcase study was conducted to prove the superiority of the designed learning scheme. The findings have demonstrated the ability of REDL in providing a meaningful feature space in each new round. Compared to relevant studies in the field, REDL algorithms brought more insights into deeper feature space than the aforementioned standard deep networks (i.e., CNNs, LSTMs, DBNs, etc.). Therefore, we expect that these developments will contribute to the shaping of a new era of representation learning and feature mapping. Additionally, regarding future prospects, the model, in this case, follows expert knowledge when giving a decision on the AULC and determining stopping criteria. So, one of the main directions will be addressing the topic of automatic stopping criteria decision-making to make the model more user-friendly. Additionally, REDL rules suffer from difficulties in finding the appropriate initial mappings allowing better AULC convergence. In this case, it is worth seeking better strategies to initialize the REDL for first rounds.

## Figures and Tables

**Table 1:** REDL training and evaluation performances for 20 rounds.

Rounds	Training				Testing				Challenge			
	RMSE	MAPE	MAE	Score	RMSE	MAPE	MAE	Score	RMSE	MAPE	MAE	Score
**1**	0.033159330	0.023722719	0.0072821542	0.92002690	0.056732137	0.049719792	0.015370994	0.81936848	0.15683731	0.12733930	0.041095521	0.63273019
**2**	0.030636718	0.027180761	0.0083214669	0.91627026	0.044685747	0.041781113	0.012895246	0.84617543	0.16161914	0.13094915	0.042248707	0.62413299
**3**	0.029612046	0.027371462	0.0083745476	0.90588462	0.048842221	0.046712078	0.014405440	0.82550979	0.16831230	0.13698862	0.044194922	0.60835212
**4**	0.026535871	0.026079915	0.0079731895	0.91825724	0.041289769	0.039362255	0.012127122	0.85150087	0.16477385	0.13238907	0.042694196	0.62304616
**5**	0.024537437	0.022679042	0.0069310660	0.93151605	0.038881902	0.036123350	0.011124481	0.86324179	0.16201790	0.13170822	0.042486887	0.62316668
**6**	0.033165578	0.031330664	0.0095755085	0.92274129	0.044238463	0.041489307	0.012783380	0.86261362	0.15949211	0.12608598	0.040625986	0.64582580
**7**	0.032748155	0.030896310	0.0094424970	0.92442864	0.043780886	0.041123148	0.012671057	0.86381602	0.15723903	0.12411878	0.039992537	0.65063918
**8**	0.034674119	0.032917842	0.010060941	0.91699874	0.046032194	0.043506488	0.013408389	0.85589385	0.15597044	0.12381225	0.039899029	0.65067977
**9**	0.036812291	0.034490943	0.010541267	0.91538703	0.045230269	0.042156879	0.012994763	0.86243391	0.15562411	0.12388387	0.039926790	0.64989793
**10**	0.038449079	0.036796127	0.011248115	0.92546928	0.038799010	0.034231789	0.010558312	0.89724779	0.15354408	0.11871769	0.038240638	0.66631281
**11**	0.030228008	0.028651439	0.0087593384	0.93371415	0.033681050	0.028362917	0.0087410798	0.89927536	0.15525524	0.12172227	0.039238192	0.65361106
**12**	0.032157868	0.029493138	0.0090174973	0.93095386	0.035147570	0.029136907	0.0089802667	0.89742321	0.15539356	0.12235874	0.039448339	0.65135765
**13**	0.028446790	0.025741093	0.0078710560	0.95874411	0.023056934	0.016441997	0.0050649284	0.94445515	0.14934975	0.11364102	0.036610916	0.67830050
**14**	0.029017091	0.025852766	0.0079071084	0.95863509	0.024963899	0.016794592	0.0051728417	0.94394165	0.14794481	0.11261426	0.036273293	0.68181121
**15**	0.034767214	0.031789653	0.0097222570	0.91922754	0.039343216	0.033002790	0.010168388	0.88278496	0.16327339	0.13169754	0.042422596	0.63577491
**16**	0.034160808	0.031082043	0.0095043415	0.90920287	0.046693861	0.040716857	0.012543668	0.85346359	0.16932282	0.13884565	0.044748176	0.61368066
**17**	0.043039709	0.040804420	0.012477824	0.91384393	0.045476168	0.039554272	0.012190895	0.88305718	0.15965536	0.12794819	0.041164067	0.65377641
**18**	0.040491357	0.038220614	0.011686840	0.91653556	0.047037777	0.041497927	0.012789562	0.87469977	0.15946926	0.13037665	0.041966382	0.64416891
**19**	0.035089344	0.034137890	0.010436657	0.94700193	0.038705546	0.036022492	0.011100662	0.90433884	**0.14338417**	**0.11235938**	**0.036145385**	**0.69007659**
**20**	**0.011156123**	**0.010599943**	**0.0032384545**	**0.98884547**	**0.017378684**	**0.015931116**	**0.0049001793**	**0.93605882**	0.14608298	0.11653841	0.037583560	0.66352206

## Data Availability

Not applicable.
